# Supplementary material for: Heat adaptation of phage T7 under an extended genetic code
Source: Virus Evol. 2021 Dec 1;7(2):veab100. doi: 10.1093/ve/veab100 (PMC8923235; doi:10.1093/ve/veab100)
Supplement: veab100_Supp [file veab100_supp.zip › Sequencing_Analysis.zip!/Sequencing_Analysis/A2/evidence/JC_48.html]

BRESEQ :: Evidence


| New junction evidence | | | | | | | | | | | |
| --- | --- | --- | --- | --- | --- | --- | --- | --- | --- | --- | --- |
|  | | seq id | position | reads (cov) | reads (cov) | score | skew | freq | annotation | gene | product |
| \* | ? | NC\_001604.1 | = 8253 | 443 (0.970) | 7 (0.020) | 7/584 | 20.4 | 1.4% | intergenic (–/–) | *–/–* | –/– |
|  | ? | NC\_001604.1 | 8503 = | 536 (1.210) |  |  |  |  | intergenic (–/–) | *–/–* | –/– |
| Rejected: Coverage evenness skew score above cutoff. | | | | | | | | | | | |
| Rejected: Frequency below/above cutoff threshold. | | | | | | | | | | | |

`GTTCGCCGTGCTGACCGTTCAATCGTATGTGCGAGCGAGCGCCGAGCTAAGATACCTCTTATTGGTAACACAGTTCCTTTGGCACCGAGCGTCCACATCATTATCACCCGTGGTGACTTTGAGAAAGCAATAGACAAGAAACGTCCGGTTCTTAGTGTGGCAGTGACCCGCTTCCCGTTCGTCCGTCTGTTACTCAAACGAATCAAGGAGGTGTTCTGATGGGACTGTTAGATGGTGAAGCCTGGGAAAAAGAAAACCCGCCAGTACAAGCAACTGGGTGTATAGCTTGCTTAGAGAAAGATGACC‑‑‑‑‑‑‑‑‑‑‑‑‑‑‑‑‑‑‑‑‑‑‑‑‑‑‑‑‑‑‑‑‑‑‑‑‑‑‑‑‑‑‑‑‑‑‑‑‑‑‑‑‑‑‑‑‑‑‑‑‑‑‑‑‑‑‑‑‑‑‑‑‑‑‑‑‑‑‑‑‑‑‑‑‑‑‑‑‑‑‑‑‑‑‑‑‑‑‑‑‑‑‑‑‑‑‑‑‑‑‑‑‑‑‑‑‑‑‑‑‑‑‑‑‑‑‑‑‑‑‑‑‑‑‑‑‑‑‑‑‑‑‑‑‑‑‑‑‑‑‑‑‑‑‑‑‑‑‑‑‑‑‑‑‑‑‑‑‑‑‑‑‑‑‑‑‑‑‑‑‑‑‑‑‑‑‑‑‑‑‑‑‑‑‑‑‑‑‑‑‑‑‑‑‑‑‑‑‑‑‑‑‑‑‑‑‑‑‑‑‑‑‑‑‑‑‑‑‑‑‑‑‑‑‑‑‑‑‑‑‑‑‑‑‑‑‑‑‑‑‑‑‑‑‑‑‑‑‑‑‑‑‑‑‑‑‑‑‑‑‑‑‑‑‑‑‑‑‑‑‑‑‑‑‑‑‑‑‑‑‑‑‑‑‑‑‑‑‑‑  >  NC_001604.1/7948‑8253`  
`‑‑‑‑‑‑‑‑‑‑‑‑‑‑‑‑‑‑‑‑‑‑‑‑‑‑‑‑‑‑‑‑‑‑‑‑‑‑‑‑‑‑‑‑‑‑‑‑‑‑‑‑‑‑‑‑‑‑‑‑‑‑‑‑‑‑‑‑‑‑‑‑‑‑‑‑‑‑‑‑‑‑‑‑‑‑‑‑‑‑‑‑‑‑‑‑‑‑‑‑‑‑‑‑‑‑‑‑‑‑‑‑‑‑‑‑‑‑‑‑‑‑‑‑‑‑‑‑‑‑‑‑‑‑‑‑‑‑‑‑‑‑‑‑‑‑‑‑‑‑‑‑‑‑‑‑‑‑‑‑‑‑‑‑‑‑‑‑‑‑‑‑‑‑‑‑‑‑‑‑‑‑‑‑‑‑‑‑‑‑‑‑‑‑‑‑‑‑‑‑‑‑‑‑‑‑‑‑‑‑‑‑‑‑‑‑‑‑‑‑‑‑‑‑‑‑‑‑‑‑‑‑‑‑‑‑‑‑‑‑‑‑‑‑‑‑‑‑‑‑‑‑‑‑‑‑‑‑‑‑‑‑‑‑‑‑‑‑‑‑‑‑‑‑‑‑‑‑‑‑‑‑‑‑‑‑‑‑‑‑‑‑‑‑‑‑‑‑agatgaccAGCCTTCCGCTGAGACAATCGAAGGTGTCACTAAGCCTTCCCACTACATGCTGTTTGACGACATTGAGGCTATCGAAGTGATTGCTCGTTCAATGACCGTTGAGCAGTTCAAGGGATACTGCTTCGGTAACATCTTAAAGTACAGACTACGTGCTGGTAAGAAGTCAGAGTTAGCGTACTTAGAGAAAGACCTAGCGAAAGCAGACTTCTATAAAGAACTCTTTGAGAAACATAAGGATAAATGTTATGCATAACTTCAAGTCAACCCCACCTGCCGACAGCCTATCTGATGACTTCACA  >  NC_001604.1/8503‑8802`  

                                                                                                                                                                                                                                                                                                                                                                                                                                                                                                                                                                                                                                
`tgtataagagacagCCGTTCAATCGTATGTGCGAGCGAGCGCCGAGCTAAGATACCTCTTATTGGTAACACAGTTCCTTTGGCACCGAGCGTCCACATCATTATCACCCGTGGTGACTTTGAGAAAGCAATAGACAAGAAACGTCCGGTTCTTAGTGTGGCAGTGACCCGATTCCCGTTCGTCCGTCTGTTACTCAAACGAATCAAGGAGGTGTTCTGATGGGACTGTTAGATGGTGAAGCCTGGGAAAAAGAAAACCCGCCAGTACAAGCAACTGGGTGTATAGCTTGCTTAGAGAAAGA                                                                                                                                                                                                                                                                                                                   <  1:543775/287‑1`  
  `TCGCTGTGCTGACCGTTCAATCGTATGTGCGAGCGAGCGCCGAGCTAAGATACCTCTTATTGGTAACACAGTTCCTTTGGCACCGAGCGTCCACATCATTATCACCCGTGGTGACTTTGAGAAAGCAATAGACAAGAAACGTCCGGTTCTTAGTGTGGCAGTGACCCGCTTCCCGTTCGTCCGTCTGTTACTCAAACGAATCAAGGAGGTGTTCTGATGGGACTGTTAGATGGTGAAGCCTGGGAAAAAGAAAACCCGCCAGTACAAGCAACTGGGTGTATAGCTTGCTTAGAGAAAGATG                                                                                                                                                                                                                                                                                                                 >  1:816502/1‑301`  
  `TCGCCGTGCTGACCGTTCAATCGTATGTGCGAGCGAGCGCAGAGCTAAGATACCTCTTATTGGTAACACAGTTCCTTTGGCACCGAGCGTCCACATCATTATCACCCGTGGTGACTTTGAGAAAGCAATAGACAAGAAACGTCCGGTTCTTAGTGTGGCAGTGACCCGCTTCCCGTTCGTCCGTCTGTTACTCAAACGAATCAAGGAGGTGTTCTGATGGGACTGTTAGATGGTGAAGCCTGGGAAAAAGAAAACCCGCCAGTACAAGCAACTGGGTGTATAGCTTGCTTAGAGCAAGATG                                                                                                                                                                                                                                                                                                                 >  1:432173/1‑301`  
    `GCCGTGCTGACCGTTCAACCGTATGTGCGAGCGAGCGCCGAGCTAAGATACCTCTTATTGGTAACACAGTTCCTTTGGCACCGAGCGTCAACATCATTATCACCCGTGGTGACTTTGAGAAAGCAATAGACAAGAAACGTCCGGTTCTTAGTGTGGCAGTAACCCGCTTCCCGTTCGTCCGTCTGTTACTCAAACGAATCAAGGAGGTGTTCTGATGGGACTGTTAGATGGTGAAGCCTGGGAAAAAGAAAACCCGCCAGTACAAGCAACTGGGTGTATAGCTTGCTTAGAGAAAGATGAC                                                                                                                                                                                                                                                                                                               <  1:754870/301‑1`  
     `CCGTGCTGACCGTTCAATCGTATGTGCGAGCGAGCGCCGAGCTAAGATACCTCTTATTGGTAACACAGTTCCTTTGGCACCGAGCGTCCACATCATTCTCACCCTTGGTGACTTTGAGAAAGCAATCGACAAGACACGTCCGTTTCTTAGTGTGGCAGTGACCCGCTTCCCGTTCGTCCGTCTGTTACTCAACCGAATCAAGGAGGTGTTCTGTTGGTCCTGTTAGTTGGTGAAGCCTGTGAAAAAGAAAACCCGCCAGTACAAGCACCTGGTTGTATAGCTTGCTTAGAGAAAGATGACC                                                                                                                                                                                                                                                                                                              >  1:564737/1‑301`  
       `GTGCTTAACGTTAAATGGGATGTGAGAGAGAGAGGAGAGATAAGATACCTCTTATTGGTAACAAAGTTCCTTTGGCATCGAGCGTACACAGCATGATCAACCGTGGTGACTTTGAGAAAGCAATAGACAAGAACCGTCCGGTTCTTAGTGTGGCAGTGACCCGCTTCCCGTTCGTCCGTCTGTTACTCAAACGAATCAAGGAGGTGTTCTGATGGGACTGTTAGATGGTGAAGCCTGTGAAAAAGAAAACCAGCCAGTACAAGCAACTGGGTGTATAGCTTGCTTAGAGAAAGATGACagt                                                                                                                                                                                                                                                                                                            <  1:783181/301‑4`  
                     `ATCGTATGTGCGAGCGAGCTCCGAGCTAAGATACCTCTTATTGGTAACACAGTTCCTTTGGCACCGAGCGTCCACATCATTATCACCCGTGGTGACTTTGAGAAAGCAATAGACAAGAAACGTCCTGTTCTTAGTGTGGCAGTGACCCGCTTCCCGTTCGTCCGTCTGTTACTCAAACGAATCAAGGAGGTGTTCTGATGGGACTGTTAGATGGTGAAGCCTGGGAAAAAGAAAACCCGCCAGTACAAGCAACTGGGTGTATAGCTTGCTTAGAGAAAGATGACCAGCCTTCCGCTGAGAC                                                                                                                                                                                                                                                                                              >  1:349271/1‑301`  
                             `TGCGAGCGAGCGCCGAGCTAAGATACCTCTTATTGGTAACACAGTTCCTTTGGCACCGAGCGTCCACATCATTATCACCCGTGGTGACTTTGAGAAAGCAATAGACAAGTAACGTCCGGTTCTTAGTGTGGCAGTGACCCGCTTCCCGTTCGTCCGTCTGTTACTCAAACGAATCAAGGAGGTGTTCTGATGGGACTGTTAGATGGTGAAGCCTGGGAAAAAGAAAACCCGCCAGTACAAGCAACTGGGTGTATAGCTTGCTTAGAGAAAGATGACCAGCCTTCCGCTTAGACAATCGAAG                                                                                                                                                                                                                                                                                      >  1:248320/1‑301`  
                                                     `ACCTCTTATTGGTAACACAGTTCCTTTGGCACCGAGCGTCCACATCATTATCACCCGTGGTGACTTTGATAAAGCAATAGACAAGAAACGTCCGGTTCTTAGTGTGGCAGTGACCCGCTTCCCGTTCGTCCGTCTGTTACTCAAACGAATCAAGGAGGTGTTCTGATGGGACTGTTAGATGGTGAAGCCTGGGAAAAAGAAAACCCGCCAGTACAAGCAACTGGGTGTATAGCTTGCTTAGAGAAAGATGACCAGCCTTCCACTGAGACAATCGAAGGTGTCACGAAGCCTGCCCACTACA                                                                                                                                                                                                                                                              >  1:742066/1‑301`  
                                                                   `ACACAGTTCCTTTGGCACCGAGCGTCCACATCATTATCACCCGTGGTGACTTTGAGAAAGCAATAGACAAGAAACGTCCGGTTCTTAGTGTGGCAGTGACCCGCTTCCCGTTCGTCCGTCTGTTACTCAAACGAATCAAGGAGGTGTTCTGATGGGACTGTTAGATGGTGAAGCCTGGGAAAAAGAAAACCCGCCAGTACAAGCAACTGGGTGTATAGCTTGCTTAGAGAAAGATGACCAGCCTTCCGCTGAGACAATCGAAGGTGTCACTAAGCCTTCCCACTACATGCTGTTTGACGAC                                                                                                                                                                                                                                                >  1:767494/1‑301`  
                                                                                                                                                                                        `GTCTGTTACTCAAACGAATCAAGGAGGTGGTCTGATGGGACTGTTAGATGGTGAAGCCTGGGAAAAAGAAAACACGCCAGTACAAGCAACTGGGTGTATAGCTTGCTTAGAGAAAGATGACCAGCCTTCCGCTGAGACAATCGAAGGTGTCACTAAGCCTTCCCACTACATGCTGTTTGACGACATTGAGGCTATCGAAGTGATTGCTCGTTCAATGACCGTTGAGCAGTTCAAGGGATACTGCTTCGGTAACATCTTAAAGTACAGACTACGTGCTGGTAAGAAGTCAGAGTTAGCGTAC                                                                                                                           <  1:348751/301‑1`  
                                                                                                                                                                                                                 `GGTGTTCTGATGGGACTGTTAGATGGTGAAGCCTGGGAAAAAGAAAACCCGCCAGTACAAGCAACTGGGTGTATAGCTTGCTTAGAGAAAGATGACCAGCCTTCCGCTGAGACAATCGAAGGTGTCACTAAGCCTTCCCACTACATGCTGTTTGACGACATTGAGGCTATCGAAGTGATTGCTCGTTCAATGACCGTTGAGCAGTTCAAGGGATACTGCTTCGGTAACATCTTAAAGTACAGACTACGTGCTGGTAAGAAGTCAGAGTTAGCGTACTTAGAGAAAGACCTAGCGAAAGCAG                                                                                                  >  1:57463/1‑301`  
                                                                                                                                                                                                                                                                                   `GGGTGTATAGCTTGCTTAGAGAAAGATGACCAGCCTTCCGCTGAGACAATCGAAGGTGTCACTAAGCCTTCCCACTACATGCTGTTTGACGACATTGAGGCTATCGAAGTGATTGCTCGTT‑AATGACCGTTGAGCAGTTCAAGGGATACTGCTTCGGTAACATCTTAAAGTACAGACTACGTGCTGGTAAGAAGTCAGAGTTAGCGTACTTAGAGAAAGACCTAGCGAAAGCAGACTTCTATAAAGAACTCTTTGAGAAACATAAGGATAAATGTTATGCATAACTTCAAGTCAACCCCAC                               >  1:409290/1‑301`  
                                                                                                                                                                                                                                                                                               `ggagatgtgtataagagaCAGCCTTCCGCTGAGACAATCGAAGGTGTCACTAAGCCTTCCCACTACATGCTGTTTGACGACATTGAGGCTATCGAAGTGATTGCTCGTTCAATGACCGTTGAGCAGTTCAAGGGATACTGCTTCGGTAACATCTTAAAGTACAGACTACGTGCTGGTAAGAAGTCAGAGTTAGCGTACTTAGAGAAAGACCTAGCGAAAGCAGACTTCTATAAAGAACTCTTTGAGAAACATAAGGATAAATGTTATGCATAACTTCAAGTCAACCCCACCTGCCGACAGC                    <  1:127272/283‑1`  
                                                                                                                                                                                                                                                                                                           `GATGACCAGCCTTCCGCTGAGACAATCGAAGGTGTCACTAAGCCTTCCCACTACATGCTGTTTGACGACATTGAGGCTATCGAAGTGATTGCTCGTTCAATGACCGTTGAGCAGTTCTAGGGATACTGCTTCGGTAACATCTTATAGTACAGACTACGTGCTGGTAAGAAGTCAGAGTTAGCGTACTTAGCGAAAGCCCTAGCGAAAGCAGACTTCTATAAAGAACTCTTTGAGAAACATAAGGATAAATGTTATGCATAACTTCAAGTCAACCACACCTGCCGACAGCCTATCTGATGAC        >  1:186296/1‑301`  
                                                                                                                                                                                                                                                                                                           `GATGACCAGCCTTCCGCTGAGACAATCGAAGGTGTCACTAAGCCTTCCCACTACATGCTGTTTGACGACATTGAGGCTATCGAAGTGATTGCTCGTTCAATGACCGTTGAGCAGTTCAAGGGATACTGCTTCGGTAACATCTTAAAGTACAGACTACGTGCTGGTAAGAAGTCAGAGTTAGCGTACTTAGAGAAAGACCTAGCGAAAGCAGACTTCTATAAAGAACTCTTTGAGAAACATAAGGATAAATGTTATGCATAACTTCAAGTCAACCCCACCTGCCGACAGCCTATCTGATGAC        <  1:591443/301‑1`  
                                                                                                                                                                                                                                                                                                           `GATGACCAGCCTTCCGCTGAGACAATCGAAGGTGTCACTAAGCCTTCCCACTACATGCTGTTTGACGACATTGAGGCTATCGAAGTGATTGCTCGTTCAATGACCGTTGAGCAGTTCAAGGGATACTGCTTCGGTAACATCTTAAAGTACAGACTACGTGCTGGTAAGAAGTCAGAGTTAGCGTACTTAGAGAAAGACCTAGCGAAAGCAGACTTCTATAAAGAACTCTTTGAGAAACATAAGGATAAATGTTATGCATAACTTCAAGTCAACCCCACCTGCCGACAGCCTATCTGATGAC        <  1:777156/301‑1`  
                                                                                                                                                                                                                                                                                                           `GATGACCAGCCTTCCGCTGAGACAATCGAAGGTGTCACTAAGCCTTCCCACTACATGCTGTTTGACGACATTGAGGCTATCGAAGTGATTGCTCGTTCAATGACCGTTGAGCAGTTCAAGGGATACTGCTTCGGTAACATCTTAAAGTACAGACTACGTGCTGGTAAGAAGTCAGAGTTAGCGTACTTAGAGAAAGACCTAGCGAAAGCAGACTTCTATAAAGAACTCTTTGAGAAACATAAGGATAAATGTTATGCATAACTTCAAGTAAACCACACCTGCCGACAGCCTATCTGATGAC        >  1:179435/1‑301`  
                                                                                                                                                                                                                                                                                                           `GATGACCAGCCTTCCGCTGAGACAATCGAAGGTGTCACTAAGCCTTCCAACTACATGCTGTTTGACGACATTGAGGCTATCGAAGTGATTGCTCGTTCAATGACCGTTGAGCAGTTCAAGGGATACTGCTTCGGTAACATCTTAAAGTACAGACTACGTGCTGGTAAGAAGTCAGAGTTAGCGTACTTAGAGAAAGACCTAGCGAAAGCAGACTTCTATAAAGAACTCTTTGAGAAACATAAGGATAAATGTTATGCATAACTTCAAGTCAACCCCACCTGCCGACAGCCTATCTGATGAC        <  1:800041/301‑1`  
                                                                                                                                                                                                                                                                                                              `GACCAGCCTTCCGCTGAGACAATCGAAGGTGTCACTAAGCCTTCCCACTACATGCTGTTTGACGACATTGAGGCTATCGAAGTGATTGCTCGTTCAATGACTGTTGAGCAGTTCAAGGGATACTGCTTCGGTAACATCTTAAAGTACAGACTACGTGCTGGTAAGAATTCAGAGTTAGCGTACTTAGAGAAAGACCTAGCGAAAGCAGACTTCTATAAAGAACTCTTTGAGAAACATAAGGATAAATGTTATGCATAACTTCAAGTCAACCCCACCTGCCGACAGCCTATCTGATGACTTC     >  1:454106/1‑301`  
                                                                                                                                                                                                                                                                                                              `GACCAGCCTTCCGCTGAGACAATCGAAGGTGTCACTAAGCCTTCCCACTACATGCTGTTTGACGACATTGAGGCTATCGAAGTGATTGCTCGTTCAATGACCGTTGAGCAGTTCAAGGGATACTGCTTCGGTAACATCTTAAAGTACAGACTACGTGCTGGTAAGAAGTCAGAGTTAGCGTACTTAGAGAAAGACCTAGCGAAAGCAGACTTCTATAAAGAACTCTTTGAGAAACATAAGGATAAATGTTATGCATAACTTCAAGTCAACCCCACCTGCCGACAGCCTATCTGATGACTTC     >  1:396861/1‑301`  
                                                                                                                                                                                                                                                                                                                `CCAGCCTTCCGCTGAGACAATCGAAGGTGTCACTAAGCCTTCCCACTACATGCTGTTTGACGACATTGAGGCTATCGAAGTGATTGCTCGTTCAATGACCGTTGAGCAGTTCAAGGGATACTGCTTCGGTATCATCTTAAAGTACAGACTACGTGCTGGTAAGAAGTCAGAGTTAGCGTACTTAGAGAAAGACCTAGCGAAAGCAGACTTCTATAAAGAACTCTTTGAGAAACATAAGGATAAATGTTATGCATAACTTCAAGTCAACCCCACCTGCCAACAGCCTATCTGATGACTTCAC   >  1:577490/1‑301`  
                                                                                                                                                                                                                                                                                                                 `CAGCCTTCCGCTGAGACAATCGAAGGTGTCACTAAGCCTTCCCACTACATGCTGTTTGACGACATTGAGGCTATCGAAGTGATTGCTCGTTCAATGACCGTTGAGCAGTTCAAGGGATACTGATTCGGTAACATCTTAAAGTACAGACTACGTGCTGGTAAGAAGTCAGAGTTAGCGTACTTAGAGAAAGACCTAGCGAAAGCAGACTTCTATAAAGAACTCTTTGAGAAACATAAGGATAAATGTTATGCATAACTTCAAGTCAACCCCACCTGCCGACAGCCTATCTGATGACTTCACA  <  1:209488/301‑1`  

                                                                                                                                                                                                                                                                                                                                                                                                                                                                                                                                                                                                                                
`GTTCGCCGTGCTGACCGTTCAATCGTATGTGCGAGCGAGCGCCGAGCTAAGATACCTCTTATTGGTAACACAGTTCCTTTGGCACCGAGCGTCCACATCATTATCACCCGTGGTGACTTTGAGAAAGCAATAGACAAGAAACGTCCGGTTCTTAGTGTGGCAGTGACCCGCTTCCCGTTCGTCCGTCTGTTACTCAAACGAATCAAGGAGGTGTTCTGATGGGACTGTTAGATGGTGAAGCCTGGGAAAAAGAAAACCCGCCAGTACAAGCAACTGGGTGTATAGCTTGCTTAGAGAAAGATGACC‑‑‑‑‑‑‑‑‑‑‑‑‑‑‑‑‑‑‑‑‑‑‑‑‑‑‑‑‑‑‑‑‑‑‑‑‑‑‑‑‑‑‑‑‑‑‑‑‑‑‑‑‑‑‑‑‑‑‑‑‑‑‑‑‑‑‑‑‑‑‑‑‑‑‑‑‑‑‑‑‑‑‑‑‑‑‑‑‑‑‑‑‑‑‑‑‑‑‑‑‑‑‑‑‑‑‑‑‑‑‑‑‑‑‑‑‑‑‑‑‑‑‑‑‑‑‑‑‑‑‑‑‑‑‑‑‑‑‑‑‑‑‑‑‑‑‑‑‑‑‑‑‑‑‑‑‑‑‑‑‑‑‑‑‑‑‑‑‑‑‑‑‑‑‑‑‑‑‑‑‑‑‑‑‑‑‑‑‑‑‑‑‑‑‑‑‑‑‑‑‑‑‑‑‑‑‑‑‑‑‑‑‑‑‑‑‑‑‑‑‑‑‑‑‑‑‑‑‑‑‑‑‑‑‑‑‑‑‑‑‑‑‑‑‑‑‑‑‑‑‑‑‑‑‑‑‑‑‑‑‑‑‑‑‑‑‑‑‑‑‑‑‑‑‑‑‑‑‑‑‑‑‑‑‑‑‑‑‑‑‑‑‑‑‑‑‑‑‑‑  >  NC_001604.1/7948‑8253`  
`‑‑‑‑‑‑‑‑‑‑‑‑‑‑‑‑‑‑‑‑‑‑‑‑‑‑‑‑‑‑‑‑‑‑‑‑‑‑‑‑‑‑‑‑‑‑‑‑‑‑‑‑‑‑‑‑‑‑‑‑‑‑‑‑‑‑‑‑‑‑‑‑‑‑‑‑‑‑‑‑‑‑‑‑‑‑‑‑‑‑‑‑‑‑‑‑‑‑‑‑‑‑‑‑‑‑‑‑‑‑‑‑‑‑‑‑‑‑‑‑‑‑‑‑‑‑‑‑‑‑‑‑‑‑‑‑‑‑‑‑‑‑‑‑‑‑‑‑‑‑‑‑‑‑‑‑‑‑‑‑‑‑‑‑‑‑‑‑‑‑‑‑‑‑‑‑‑‑‑‑‑‑‑‑‑‑‑‑‑‑‑‑‑‑‑‑‑‑‑‑‑‑‑‑‑‑‑‑‑‑‑‑‑‑‑‑‑‑‑‑‑‑‑‑‑‑‑‑‑‑‑‑‑‑‑‑‑‑‑‑‑‑‑‑‑‑‑‑‑‑‑‑‑‑‑‑‑‑‑‑‑‑‑‑‑‑‑‑‑‑‑‑‑‑‑‑‑‑‑‑‑‑‑‑‑‑‑‑‑‑‑‑‑‑‑‑‑‑agatgaccAGCCTTCCGCTGAGACAATCGAAGGTGTCACTAAGCCTTCCCACTACATGCTGTTTGACGACATTGAGGCTATCGAAGTGATTGCTCGTTCAATGACCGTTGAGCAGTTCAAGGGATACTGCTTCGGTAACATCTTAAAGTACAGACTACGTGCTGGTAAGAAGTCAGAGTTAGCGTACTTAGAGAAAGACCTAGCGAAAGCAGACTTCTATAAAGAACTCTTTGAGAAACATAAGGATAAATGTTATGCATAACTTCAAGTCAACCCCACCTGCCGACAGCCTATCTGATGACTTCACA  >  NC_001604.1/8503‑8802`

  

Alignment Legend

Aligned base mismatch/match (shaded by quality score): `ATCG/ATCG` `< 3 ≤` `ATCG/ATCG` `< 11 ≤` `ATCG/ATCG` `< 26 ≤` `ATCG/ATCG` `< 36 ≤` `ATCG/ATCG` `< 38 ≤` `ATCG/ATCG`

Unaligned base: `atcg`    Masked matching base: `atcg`    Alignment gap: `‑`    Deleted base: `‑`

Reads not counted as support for junction

read\_name Not counted due to insufficient overlap past the breakpoint.

read\_name Not counted due to not crossing MOB target site duplication.
